# Immunotherapeutic Strategies for Alzheimer's Disease Treatment

**DOI:** 10.1100/tsw.2009.99

**Published:** 2009-09-01

**Authors:** Beka Solomon

**Affiliations:** Department of Molecular Microbiology and Biotechnology, George S. Wise Faculty of Life Sciences, Tel-Aviv University, Tel Aviv, Israel

**Keywords:** Alzheimer’s disease, amyloid-β, amyloid plaque, inflammation, immunotherapy, neuropathology

## Abstract

Naturally occurring antibodies against amyloid-β peptides have been found in human cerebrospinal fluid and in the plasma of healthy individuals, but were significantly lower in Alzheimer's disease (AD) patients, suggesting that AD may be an immunodeficient disorder. The performance of anti-amyloid-β antibodies in transgenic mice models of AD showed that they are delivered to the central nervous system, preventing and dissolving amyloid-β plaques. Moreover, these antibodies protected the mice from learning and age-related memory deficits. Active and/or passive immunization against the amyloid-β peptide has been proposed as a method for preventing and/or treating AD. Immunotherapy represents fascinating ways to test the amyloid hypothesis and offers genuine opportunities for AD treatment, but requires careful antigen and antibody selection to maximize efficacy and minimize adverse events.

## INTRODUCTION

The pathology of Alzheimer's disease (AD) is characterized primarily by extracellular plaques and intracellular neurofibrillary tangles[1]. Plaques are composed mainly of amyloid-β (Aβ) peptides, whereas tangles are composed of the cytoskeletal protein tau. The relationship between these lesions and the disease process has long been debated. The current dominant theory of AD etiology and pathogenesis related to the amyloid cascade hypothesis[2] states that overproduction of Aβ peptides (AβPs), or failure to clear these peptides, leads to AD, primarily through amyloid deposition, which is presumed to be involved in neurofibrillary tangle formation; these lesions are then associated with cell death, which is reflected in memory impairment, the hallmark of this dementia. Over the last 10 years, the amyloid cascade hypothesis has gained strength through the observation that AD-causing mutations were identified in the Aβ precursor protein (AβPP) and in the presenilin genes[3,4].

AβP is a normal soluble metabolite of ~4 kDa that is produced by processing a large transmembrane glycoprotein, called amyloid precursor protein (APP), by β- and γ-secretase[5]. The pathological conditions and mechanisms that transform soluble AβP into the fibrillary, toxic, β-sheet form, either low-molecular-weight oligomers or insoluble fibrils found in plaques and vessels of AD patients, are not yet completely understood. However, it is clear that the same amino acid sequence of AβP can have distinct conformations that lead to either the toxic soluble or fibrillar state of the peptide.

*In vitro* experimental data have shown that antibodies could disassemble amyloid fibrils and protect neurons from Aβ toxicity, suggesting possible benefits in AD treatment. Various active and passive immunizations that target amyloid-β proved capable of providing significant therapeutic benefits in behavior and cognition when assessed in transgenic (Tg) mouse models of AD. Although the first clinical trial to employ an active immunization protocol was halted for safety reasons, it indicated clinical efficacy of the approach in humans.

## IMMUNOLOGICAL CONCEPT OF AD IMMUNOTHERAPY

### The Immune System Participation in AD Pathogenesis

The immune system appears to participate in AD pathogenesis. Tolerance to a self-antigen and immune down-regulation associated with aging may blunt the immune response to Aβ[6,7,8,9,10,11,12]. There is evidence for partial tolerance against Aβ in APP Tg mice[6]. Chronic exposure of the immune system to Aβ in humans and mouse models might lead to hyporesponsiveness in terms of cellular and humoral immune responses to Aβ itself, which could contribute to the disease process[6].

Indeed, the titer of anti-AβP42 antibodies in serum from individuals with and without late-onset AD, measured using an enzyme-linked immunosorbent assay (ELISA)[13], showed that IgG titer of anti-AβP42 peptide antibodies was significantly higher in serum from elderly controls than from AD patients. However, the low titer of anti-AβP42 antibodies in AD patients does not reflect the well-established, age-associated defect in the antibody response to most protein antigens, as there was no positive correlation between the serum titer of anti-AβP42 antibodies and anti-influenza hemagglutinin antibodies induced by influenza vaccine in elderly humans. The lower titer of serum anti-AβP42 peptide antibodies in AD patients may reflect specific impairment of helper T-cell activity for B cells that produce anti-AβP42 peptide antibodies or tolerance to self-antigen.

Human B lymphocytes have the capacity to produce anti-AβP antibodies[14]. A recent study showed that plasma anti-AβP antibodies that bind to aggregated AβP were significantly lower in people with AD than in healthy controls, while there was no difference in anti-AβP antibodies binding to AβP monomers[15]. Therefore, natural antibodies to aggregated AβP may have great importance against AD pathology.

### Modulation of Amyloid-β Conformation

Many investigators have studied the propensity of AβP or its fragments to assemble into soluble/insoluble aggregates[16,17,18,19]. AβP can exist in different conformations, depending on the secondary structure adopted by the N-terminal domain under various environmental conditions[17,18]. The N-terminal domain contains sequences that permit the existence of a dynamic equilibrium between the α-helix and the β-strand conformations. The perturbations of the equilibrium of the conformational states of AβP can be caused by local pH changes, alterations of environmental hydrophobicity, or binding of other proteins[19]. The dependence of AβP polymerization on peptide-peptide interactions to form a β-pleated sheet fibril and the stimulatory influence of other proteins on the reaction suggest that amyloid formation may be subject to modulation.

The availability of monoclonal antibodies (mAbs) has facilitated the understanding of how highly specific antigen-antibody interactions affect antigen stability and conformation. The complementary interaction between regions of the antibody and its antigen confer high specificity and stability to the immunocomplex formed. Antibodies known as reporting probes for the detection of antigens are able to play an active role in inducing changes in the conformation of the antigen molecule. Antibody-antigen interactions involve conformational changes that can range from insignificant to considerable. Binding of high-affinity mAbs to regions of high flexibility and antigenicity may alter the molecular dynamics of the whole antigen and may induce structural rearrangements in the molecular edifice[20,21,22,23,24]. Like the ubiquitous chaperones, mAbs raised against specific native antigens may assist in its refolding[25,26,27] by recognizing incompletely folded epitopes and inducing their native conformation. Appropriate mAbs interact at strategic sites where protein aggregation is initiated, stabilizing the protein, preventing further aggregation, and resolubilizing already-formed protein aggregates[25].

We investigated a large panel of mAbs against various regions of AβP and found that mAbs that target the N-terminal regions of AβP exhibit antiaggregating properties[28,29,30]. Binding of such antibodies to aggregated AβP interfered with noncovalent interactions between the amyloid fibrils and led to deterioration of amyloid fibrillar assembly. Disaggregation, as well as prevention of amyloid formation, was found to be dependent on the location of the epitopes on Aβ and on the binding characteristics of the respective antibodies.

Using the phage-peptide library composed of filamentous phage-displaying random combinatorial peptides, we defined the GLU-PHE-ARG-HIS (EFRH residues located at positions 3 to 6 of the N-terminal AβP) as the epitope of the antiaggregating antibodies studied[31,32]. Locking of the EFRH epitope by the respective antibodies was found to modulate the dynamics of aggregation, as well as to resolubilize already-formed aggregates, confirming its key role in modulation of conformational changes of the whole AβP molecule.

Identification of the "aggregating epitopes" as strategic positions related to sites where protein aggregation is initiated, and preparing antibodies against these regions, became the basis of the immunological concept for treatment of so-called conformational diseases, such as AD.

## IMMUNOLOGICAL STRATEGIES FOR PREVENTION AND/OR REDUCTION OF AMYLOID PLAQUES IN TRANSGENIC MICE MODEL OF AD

With the development of AD animal models (for review see [33]), the immunological concept in the treatment of conformational diseases became a therapeutic approach to stimulate clearance of brain Aβ plaques, either as active or passive immunization[34,35]. Active immunization approaches employ AβP epitopes and/or immunogenic AβP conjugates, as well as various routes of administration and types of adjuvants

Passive immunization approaches include antibodies or antibody fragments directed against specific AβP epitopes. This procedure provides antibodies directly to the body, rather than requiring a self-immunological response.

### Active Immunization Approaches

Immunization with human synthetic Aβ42 of AD transgenic mice that harbor a mutant version of human APP770 (V717F) was shown to produce high serum antibody titers against Aβ42 (1:10,000), and inhibited the formation of amyloid plaques and associated histopathologic lesions[36].

Weiner and colleagues showed that mucosal administration of human Aβ40 may affect neuropathological lesions, accompanied by a 52% decrease in brain Aβ42 levels[37]. Mucosal immunization with Aβ1-42 induces antibodies to Aβ and T cells that may have regulatory properties. As almost all the studied human Aβ-reactive T-cell lines also showed a $T_h2$ phenotype, it is possible that mucosal immunization that preferentially induces $T_h2$ or $T_h3$ responses could boost this lineage and enhance clearance of Aβ by stimulating Aβ antibody production, and by modulating microglial activation at sites of Aβ plaques, with a minimal risk of harmful T-cell response in the central nervous system (CNS)[38].

Since administration of human Aβ42 in AD patients may induce adverse effects, the Wisniewski group believed that Aβ42 immunization in humans may be unsafe because this peptide may cross the blood brain barrier (BBB) and form toxic fibrils, and proposed sequences of Aβ devoid of fibrillogenic properties as antigens[39].

Immunization of 10-month-old Tg2576 mice with soluble AβP1-30 produced significant antibody titers specific to the first 16 residues of AβP. After 7 months of immunization, the 18-month-old mice showed 89 and 81% reduction in cortical and hippocampal brain amyloid burden, respectively.

Another derivative of the AβP1-16 peptide sequence was synthesized by covalently attaching two palmitoyl residues at each end of the peptide and subsequently reconstituting it in liposomes[40]. Vaccination of the animals with the bipalmitoyl derivative of AβP1-16 elicited strong antibody titers within 10 weeks after the first inoculation. The immunized mice sera were able to dissolve *in vitro* up to 80% of preformed Aβ42 aggregates.

We developed an immunization procedure for the production of effective antiaggregating AβP antibodies based on filamentous phages displaying, on their surface, the EFRH peptide located between amino acids 3 to 6 of AβP as antigen. Results obtained from experiments in transgenic animals supported the *in vitro* studies[29,30,31]. Sixteen-month-old APP[V717I] Tg mice were immunized with the EFRH phage for a 5-month period. Brain amyloid burden was significantly reduced in the immunized mice that developed anti-AβP titers of at least 1:100, indicating that a relatively low antibody titer may reduce brain amyloid load[41].

In an additional set of experiments, the cognitive behavior of the animals treated with phage EFRH was evaluated by testing the spatial and temporal navigation of each in the Morris Water Maze (MWM). A considerable improvement in their cognitive functions was obtained, dependent on the treatment. Regardless of the exact mechanism, it is clear that reversal of memory impairment is attributable to the effect of EFRH immunization. Mice with relatively high levels of antibodies to EFRH behaved in a manner similar to the nontransgenic mice in the MWM test. In spite of the fact that plaque load is the most widely used pathological outcome measured in the preclinical assessment of anti-Aβ treatments in the present study, only moderate correlation between amyloid burden and improvement in water maze performance (path length) was found[42].

Recently, Lemere and colleagues performed immunization of transgenic mice carrying human amyloid precursor protein, familial AD ($hAPP_{FAD}$) mice with antigens based on AβP1-15, which resulted in high anti-Aβ titers of noninflammatory T-helper 2 isotypes (IgG1 and IgG2b), a lack of splenocyte proliferation against full-length Aβ, significantly reduced Aβ plaque load, and lower cerebral Aβ levels. In addition, immunized $hAPP_{FAD}$ mice showed improved acquisition of memory compared with vehicle controls in a reference-memory MWM behavior test that approximately correlated with anti-Aβ titers[43].

## Passive Immunization Approaches

Peripheral administration of antibodies directed against AβP was reported to clear amyloid burden in the brain of PDAPP Tg mice. Some of these antibodies enter the brain, bind to plaques, and trigger microglial-dependent, clearance amyloid plaques. Of the antibodies tested, only mAbs 10D5, 3D6, and polyclonal anti-AβP1-42, directed to the N-terminal regions of AβP, demonstrated antiaggregating properties *in vivo*[44]. Peripheral administration of antibodies against AβP was sufficient to reduce brain amyloid burden in the PDAPP Tg mice. In contrast, mAbs 16C11, 21F12, and the control antibody TM2a, directed to other regions of AβP, were inactive. This result is consistent with the inability of these two antibodies to decorate plaques after *in vivo* administration and explains their inability to trigger plaque clearance. These *in vivo* data confirm previous *in vitro* data[28,29,30] that only antibodies directed to the "strategic" epitopes involved in the aggregation process, such as EFRH, exhibit so-called "chaperone-like" properties in dissolving amyloid plaques.

However, Pfeifer et al.[45], using APP23-Tg mice, suggested that a high concentration of antibodies against the N-terminus of AβP, which recognize amyloid present in the cerebral vasculature, might predispose patients with cerebral amyloid angiopathy to microhemorrhages and hemorrhagic stroke.

Passive immunization with mAb 266, which binds to the central part of AβP, reduces brain Aβ burden in transgenic mice[46]. Within hours after intravenous injection of mAb 266 to the mice, there was a 1,000-fold increase in the concentration of AβP in plasma, suggesting that the antibody decreases AβP deposition, at least in part, by decreasing the transfer of AβP from plasma to the CNS and increasing its transfer from the CNS to the plasma.

Although peripheral administration of mAb 266 markedly reduced amyloid burden, it failed to bind amyloid deposits in the brain. Passive vaccination strategies improved memory performance in AD APP Tg mice. Repeated injections (six weekly), and even a single injection of mAb 266, produced marked normalization of behavioral performances in PDAPP mice without any apparent effects on amyloid burden.

The effect of the administration of BC05 antibody, developed against the carboxyl (C)-termini of AβP42(43), on the clearance of brain Aβ, caused a selective 44-fold increase in plasma Aβ42(43) and a significant increase in brain soluble Aβ42(43). Brain insoluble Aβ40 and Aβ42(43) levels were decreased by 27.3 and 31.5%, respectively. A reduction in the number of labeled plaques was observed[47].

The effect of anti-C-terminal antibodies on amyloid levels and cognitive functions was reproduced in a subsequent study in aged APP Tg mice, but adverse effects (including cerebral microhemorrhages) were observed in this study, probably because of redistribution of disaggregated Aβ from brain parenchyma to cerebral vasculature, resulting in increased cerebral amyloid angiopathy[48,49].

Recently, several anti-AβP antibodies that target conformation epitopes have been shown to bind to pathogenic aggregated Aβ forms (protofibrils, oligomers, and amyloid plaques), but have no effect on monomeric AβP and APP in AD patients over control brains[50]. Such soluble oligomers display a conformation-dependent structure common to all oligomers independent of their sequence, which suggests a shared mechanism of toxicity. Functionally, it has been found that naturally secreted oligomers inhibit hippocampal long-term potentiation *in vivo*[12]. Taken together, these results suggest that strategies aimed at treating amyloid disorders should target oligomers of Aβ. In doing so, the equilibrium between monomers and higher-order aggregates can be disrupted, resulting in neutralization of soluble, toxic species.

Passive immunotherapy of AD would require repeated administration of anti-AβP antibodies. For that reason, human anti-AβP antibodies should be used to prevent an immune response to the currently available murine monoclonal immunoglobulins. Several methods are known to obtain human anti-AβP antibodies[51,52]. One method is humanization of murine anti-AβP antibodies by replacing framework portions of the murine anti-AβP antibodies with human framework sequences using recombinant DNA technology. Alternative methods are the generation of human monoclonal anti-AβP antibodies *in vitro* by human immunoglobulin phage library display techniques, or *in vivo* by immunization of mice whose immunoglobulin loci have been replaced by human genes.

## INTRAVENOUS IMMUNOGLOBULIN

The idea of passive immunization with intravenous immunoglobulin (IVIg) arose after the discovery that AD patients had lower levels of Aβ antibodies than did normal people of the same age[15,53,54].

Affinity-purified anti-AβP antibodies from IVIg were shown to increase AβP levels in blood and decrease Aβ levels in cerebrospinal fluid (CSF) from APP Tg[55]. Furthermore, IVIg depleted of anti-Aβ antibodies had considerably less effect on AβP levels in the blood or CSF from these animals. Anti-AβP antibody separation from IVIg was described in two different studies[55,56]. Polyclonal human natural anti-AβP antibodies were isolated from a commercial preparation of IVIg. These anti-AβP antibodies were purified by loading Octagam IVIg preparation (Octapharma) on an affinity column coated with AβP1-40 and were detected by ELISA using AβP1-40–coated plate. The purified antibodies could block

AβP fibril formation, disaggregate fibrillar AβP1-40, and prevent AβP1-40–induced neurotoxicity[55]. In addition, it was demonstrated that anti-AβP antibodies could disaggregate truncated AβP25-35 and also block AβP25-35–induced neurotoxicity, suggesting that IVIg contains antibodies against not just the N-terminal of AβP, but also the middle site of the amyloid peptide. It is also possible that other activities of IVIg, unrelated to its content of anti-Aβ antibodies, such as the modulation of inflammatory and immune reactions, may complement the effects of anti-AβP antibodies on cognitive function[56].

The molecular basis for the direct and indirect effects of IVIg on Aβ clearance was investigated using the BV-2 cellular microglia line. The data show that IVIg dissolves preformed Aβ fibrils *in vitro*. IVIg increases cellular tolerance to Aβ and enhances microglial migration toward Aβ deposits, mediating phagocytosis of Aβ fibrils[57].

## PUTATIVE MECHANISMS OF AMYLOID PLAQUE REMOVAL VIA IMMUNOTHERAPY

In theory, antibodies can provide a therapeutic benefit by either acting within the CNS or by acting in the periphery[58,59]. IgGs have limited access to the brain. Only 0.1% of an intravenous dose was shown to pass via extracellular pathways through the BBB into the brain[60]. Antibody uptake to and clearance from the brain did not differ in mice overexpressing Aβ compared with wild-type mice. Indeed, on entering the brain and binding to Aβ or amyloid plaques, the local half-lives of antibodies were not observed to be prolonged in the CNS[60], suggesting that antibody fragments may have rates and mechanisms of transport via the BBB entirely different from those of full-length antibodies.

Amyloid plaque clearance via specific anti-AβP antibodies may depend on multiple mechanisms, and understanding of these mechanisms may lead to an optimized, therapeutic approach for treatment of AD patients.

### The "Microglia-Mediated Hypothesis"

The "microglia-mediated hypothesis" suggested that anti-Aβ antibodies bind to amyloid plaques and antigen-antibody complexes are cleared via Fc receptor (FcR)–mediated phagocytosis by activated microglia[44,61,62,63]. *Ex vivo* experiments demonstrated that exogenous microglia can be induced to clear amyloid plaques from brain tissue when coincubated with certain anti-Aβ antibodies, but not $F(ab')_2$ fragments of these antibodies. These findings suggest that the Fc region of the antibodies is instrumental in microglial FcR-mediated Aβ clearance[62,63]. Controlling microglial activation will be prominent among those factors that determine whether immune therapy will be successful as a treatment for AD patients.

### Immunomodulation of AβP Conformation

Antibodies were able to enter the CNS, decorate plaques, and induce clearance of pre-existing amyloid. Small amounts of such antibodies that cross the BBB (0.1% of serum levels) might be sufficient to attenuate the further aggregation of these species into fibrillar Aβ dense-cored plaques[61]. We previously described that antibodies directed to the N-terminal region of AβP interfere with noncovalent interactions between Aβ fibrils and disaggregate them into an amorphous, nontoxic configuration[30], suggesting the *in vivo* mechanism of dissolving and removing amyloid plaques.

The same evidence was demonstrated, showing that antibodies directed against residues 4-10 of Aβ1-42 inhibit both fibrillogenesis and cytotoxicity, without eliciting a harmful cytotoxic T-cell response in TgCRND8 mice[64]. Consistent with these results are studies by Bard et al.[44,61], which demonstrate that plaque clearance is only seen with antibodies directed against the N-terminal region of Aβ.

Furthermore, a single administration of anti-Aβ3-6 IgG1 was effective at plaque clearance and resolution of neuritic lesions within 4 days, and lasted up to 32 days in the PDAPP mouse[65]. These results support the use of passive immunization strategies because, once plaques are cleared, neuronal morphology is restored and therefore may have a direct impact on cognitive function.

Indeed, local application of an anti-AβP N-terminal antibody 10D5 to an amyloid plaque led to its clearance[66]. Aβ deposits were visualized using thioflavine-S in living mice before and after therapeutic intervention. The data demonstrated that dense-core Aβ deposits are dissolved by application of mAb 10D5. Using this novel technique, the authors showed, for the first time, dissolution of existing Aβ deposits by antibody 10D5 in the brain of a transgenic mice model of AD.

Studies with $F(ab')_2$ fragments of anti-AβP antibodies devoid of the Fc region using *in vivo* topical application demonstrated that the mechanism does not require FcR-mediated cellular activation in plaque clearance by immunotherapy[67].

Shorter antibodies, like scFv, exhibit similar antigen-binding properties to their antigen as IgG antibodies, but have the advantage of lacking an Fc part. Thus, their use does not carry the risk of unwanted activation of the complement system or microglia, virtually eliminating the risk of induction of inflammatory adverse reactions. The use of scFv antibodies for the treatment of AD was pioneered by Frenkel et al.[68] who showed that scFvs were capable of disaggregating Aβ fibrils and preventing their toxic effects in cell culture. The ability of a single-chain antibody to dissolve already-formed Aβ fibrils confirms that only the antigen-binding site of the antibodies (Fab) was involved in modulation of amyloid-β conformation and not the Fc region, which was first demonstrated using 508F (scFv). Subsequently, the same researchers showed that scFv antibodies could cross the BBB and enter the CNS[69].

Additional recent studies have shown beneficial effects of scFvs on hallmarks of AD in animal models. ScFvs directed against Aβ17-28 decreased amyloid aggregation and eliminated the toxic effects of aggregated Aβ on the human neuroblastoma cell line, SH-SY5Y[70]. Moreover, anti-Aβ scFvs were expressed by the adenoassociated virus in brains of newborn[71] or aged[72] transgenic AD mice, ensuring widespread *in situ* expression of the scFvs. One year after the injection of the virus particles, a significant reduction in amyloid plaque burden was detected in both mouse strains, without any signs of neurotoxicity. Thus, it can be anticipated that low-molecular-weight antibody fragments have the potential for the same pharmacological efficacy in AD as full-length IgGs, but may be characterized by significantly improved product safety.

To ascertain the role of microglial FcR in Aβ immunotherapies definitively, APP Tg2576 mice bred into $FcR^{-/-}$ mice were used[73]. Data show that the microglia isolated from FcR-$\gamma^{-/-}$ mice exhibit almost no uptake of anti-Aβ immune complexes via FcR. Aggregated Aβ was readily scavenged by both FcR-$\gamma^{+/+}$ and FcR-$\gamma^{-/-}$ microglia in the absence of anti-Aβ. Thus, there did not appear to be any defects in the non-FcR-mediated Aβ uptake by microglia in the FcR-$\gamma^{-/-}$ mice.

Given that microglial cells from FcR-$\gamma^{-/-}$ mice are deficient in phagocytosis of anti-Aβ immune complexes and that there is no evidence for compensatory mechanisms enabling phagocytosis of immune complexes in FcR-$\gamma^{-/-}$ mice, these studies indicate that FcR-mediated mechanisms play little or no role in the effectiveness of Aβ immunotherapy in APP Tg2576 mice. Thus, it appears that the Fc portion of the anti-Aβ antibody required for interaction with FcR may not be necessary for Aβ immunotherapy to work[73].

## Peripheral Sink Hypothesis

A completely different, peripheral mechanism of Aβ clearance is apparently mediated by antibodies binding to linear, central epitopes within Aβ. Such antibodies act by binding to Aβ in the periphery and induce an efflux from the CNS (peripheral sink mechanism). Antibodies acting by the peripheral sink mechanism efficiently clear plaques from the brains of transgenic mice, associated with a fast, dramatic increase in plasma Aβ levels[74]. Although the current model predicts that this Aβ increase in the periphery is due to the ability of the antibody to alter the Aβ equilibrium between the CNS and periphery directly, it is also possible that an antibody with a high affinity for Aβ titrates the latter out of its relatively weak plasma protein binding and thus only indirectly influences Aβ efflux from the CNS. Lemere et al.[75] obtained similar results with active immunization in APP Tg mice. They found that anti-Aβ antibodies decreased the Aβ plaque burden by 75% in the brain, and increased serum Aβ level approximately 30 times.

## CONCLUSIONS

To summarize the presented data, AβP vaccination approaches in AD transgenic mice raised unprecedented hopes for an effective treatment of AD. On the efficacy side, there is evidence that AβP immunization in mice induces a clearance of Aβ plaques and improves associated cognitive disturbances. Amyloid plaque clearance via specific anti-Aβ antibodies may depend on multiple mechanisms, and understanding of these mechanisms may lead to an optimized, therapeutic approach for treatment of Alzheimer's patients. New insight into immunotherapy efficacy was attained using a triple transgenic model (3xTg-AD) that developed both lesions in AD-relevant brain regions[76]. The consequence of Aβ clearance on the development of tau pathology was evaluated. Aβ immunotherapy reduces not only extracellular Aβ plaques, but also intracellular Aβ accumulation and most notably leads to the clearance of early tau pathology. Therefore, the development of animal models has been essential to recent progress in the field, since numerous therapeutic ideas can now be conveniently tested in these models prior to human testing. It is likely that the ongoing, clinical trials will provide more information on modulation of immune responses towards therapeutic challenges and treatment for AD.
